# CO_2_ Emissions Inventory and Its Uncertainty Analysis of China’s Industrial Parks: A Case Study of the Maanshan Economic and Technological Development Area

**DOI:** 10.3390/ijerph191811684

**Published:** 2022-09-16

**Authors:** Jian Zhang, Jingyang Liu, Li Dong, Qi Qiao

**Affiliations:** SEPA Key Laboratory of Eco-Industry, Chinese Research Academy of Environmental Sciences, Beijing 100012, China

**Keywords:** industrial parks, CO_2_ emissions inventory, uncertainty analysis, climate change, China

## Abstract

The Chinese government has pledged to peak carbon emissions by 2030 and achieve carbon neutrality by 2060. Industrial parks are the key to achieving the carbon peak and neutrality in industrial sectors. Establishing the CO_2_ emissions inventory is the first step to achieve the carbon peak in industrial parks. In this study, a comprehensive CO_2_ emissions inventory was established for industrial parks, including three parts: energy consumption, industrial process, and waste disposal. We considered scope 1, 2, and 3 emissions and established an uncertainty analysis framework. Accordingly, scope 1 covered the emissions within the park boundary, scope 2 emissions covered those resulting from electricity and heat usage inside the boundary, and scope 3 included those indirect emissions beyond the boundary. The Maanshan Economic and Technological Development Area (MDA), a typical booming national eco-industrial park of China, was chosen for this case study. The results showed that the MDA CO_2_ emissions increased yearly, from 376,836.57 tons in 2016 to 772,170.93 tons in 2021. From the industrial structure perspective, heavy industry contributed the highest emissions. By dividing the emissions into scope 1, 2, and 3, scope 2 could be identified as the largest emissions source. In addition, we conducted inventory uncertainty analyses incorporated by activity levels, emissions factors, and unspecific factors. Overall, these results may promote the establishment of greenhouse gas accounting standards for Chinese industrial parks.

## 1. Introduction

The 2 °C temperature control target established by the Paris Agreement has necessitated urgent low-carbon development of the global economy. Nevertheless, global emissions of greenhouse gases (GHG), especially CO_2_, are still rising annually, particularly in industrial sectors. Unsurprisingly, climate change has become a matter of global concern. China has been the world’s largest CO_2_ emitter since 2006 and emitted approximately 9.9 billion tons of CO_2_ in 2019, accounting for 29.5% of global emissions [1]. In an ameliorative effort, China has been committed to achieve high-quality economic development while also reducing carbon emissions. Accordingly, in September 2020, China proposed their goal to peak CO_2_ emissions and achieve CO_2_ neutrality by 2030 and 2060, respectively. Moreover, China’s carbon emission intensity in 2020 was 48.4% lower than that in 2005, thereby fulfilling the 40–45% reduction target and essentially mitigating the rapid growth of CO_2_ emissions [2]. 

Although industry plays an imperative role in the Chinese economy, it also contributes to over 60% of the country’s total energy consumption and CO_2_ emissions [2]. Therefore, these industries have emerged as critical areas for energy conservation and emission reduction. Industrial parks (IPs) are the most important human resources supporting Chinese economic progress [3]. The State Council approved 552 development zones in 2018, including 219 national economic and development zones and 156 hi-tech industrial development parks [4]. In 2021, the GDP of the 230 national economic and development zones was 13.7 trillion yuan, accounting for 11.9% of the national GDP. Therefore, IPs, as intense development models, are significant parts of the regional economy [5]. However, the extensive development and rapid industrialization of IPs have adversely affected the local environment. Owing to the relatively concentrated industries in IPs, pollution is particularly prominent. In addition, the expansion of the park area and incorporation of new industrial projects therein has increased the pressure to control water, gas, and waste pollution in these IPs. Moreover, although IPs account for more than half of the country’s industrial output, they release nearly a third of the country’s CO_2_ [6,7]. Numerous low-carbon IPs have become the primary targets to facilitate exact scientific carbon emission reduction by deepening the industrial sector’s response to climate change and fully fostering green transformation during the ”14th Five-Year Plan” and subsequent periods. Promoting the green and low-carbon transformation of IPs is not only an inevitable requirement for the high-quality development of the park but would also aid in the establishment of the carbon peak at the park level [8]. Accordingly, quantitative calculation and elucidation of CO_2_ emissions in IPs is the first step towards analyzing the flow and uncertainty of CO_2_ in these parks. The establishment of an IP CO_2_ emissions inventory facilitates the formulation of specific carbon neutral policies and assists the government in evaluating the low-carbon performance of IPs.

Numerous studies have discussed the national [9,10], regional [11,12,13,14,15,16,17], enterprise [18], and industrial [19,20,21,22] accounting methods and frameworks. However, limited studies exist on IPs, and current accounting research on IP CO_2_ emissions has not yet produced systematic results. The established literature has adopted a consumption perspective by following the scopes defined by the World Resources Institute (WRI) and the World Business Council for Sustainable Development (WBCSD) and employing Intergovernmental Panel on Climate Change (IPCC) reference methods. According to the guidelines published by WRI and WBCSD, carbon emissions are classified in three scopes: scope 1 encompasses all direct GHG emissions in the park, sources of which include, among others, energy consumption, industrial processes, and waste disposal; scope 2 encompasses GHG emissions caused by the use of electricity and heat purchased from outside the park; and scope 3 encompasses all indirect emissions apart from those of scope 2, such as the upstream production emissions of purchased raw materials and the usage emissions of sold products. Accordingly, Liu et al. [23] and Wang et al. [24] calculated scope 1 and 2 emissions of the Suzhou industrial park. Yu et al. [25] established a self-consistent approach and framework for constructing park-level GHG emission inventories in China. These emission inventories considered both scope 1 and 2 emissions from fossil fuel combustion and electricity and heat imports, respectively. Similarly, Liu et al. [26] created a detailed GHG emissions inventory for the Beijing Economic Technological Development Area with a particular focus on scope 1 and 2 emissions, although important scope 3 emissions were also considered. Several studies have adopted a life cycle perspective on emissions accounting for industrial parks. For example, Chen et al. [27] developed an inventory to analyze the GHG emissions of a high-end IP in Beijing and divided the park’s life cycle into three phases: construction, operation, and demolition. Guo et al. [28] investigated the life cycle GHG emissions from energy use, including fuel production, transportation, and combustion, in 213 Chinese national IPs. Alternatively, Dong et al. [29] chose a hybrid life cycle assessment method to estimate the carbon footprint of a Chinese IP by considering onsite, upstream, and downstream GHG emissions.

These previous methods have significant uncertainties in terms of data collection, accounting boundaries, and methodologies that may affect the comparability of the results. In addition, most studies mainly focus on energy-related emissions, thereby failing to include a comprehensive emissions scope. Therefore, this paper aimed to establish a CO_2_ emissions accounting framework for IPs. A CO_2_ emissions inventory was established by combining the characteristics of the Maanshan Economic and Technological Development Area (MDA) from 2016 to 2021. By means of this MDA CO_2_ emission inventory, we further analyzed CO_2_ flow. Furthermore, we conducted an uncertainty analysis of the emission inventory. This CO_2_ emissions accounting framework may be applied to different types of IPs with diverse energy and economic systems. Under this framework, IPs in China can develop standardized CO_2_ emission inventories with feasible accounting methods and consistent boundaries, which ensures comparable emission results. Therefore, the results of this study provide a data basis to promote CO_2_ emissions accounting for IPs and support IP carbon peak and neutrality actions.

The remainder of this study is organized as follows: Section 2 develops a comprehensive CO_2_ emissions accounting and uncertainty analysis framework for Chinese IPs; Section 3 tests and verifies the framework by conducting an empirical study on the MDA; and Section 4 presents the conclusions and policy implications.

## 2. Materials and Methods

### 2.1. Case Park

The MDA is located in the southern part of Maanshan city and has an area of 34.47 km^2^. The MDA, which was founded in 1995, was upgraded to a national economic and technological development area in 2010. After more than two decades of development in the MDA, three dominant industries have emerged: equipment manufacturing, automotive industry, and food processing and manufacturing. The MDA is committed to building a regional industrial symbiosis system based on market mechanisms to maximize efficient use of resources, minimize pollutant emissions, and improve regional environmental and economic growth quality. The MDA industrial added value was 14.2 billion yuan in 2020, accounting for 17% of the industrial added value in Maanshan. The MDA actively develops a low-carbon economy by consolidating economic tools based on low-carbon energy, technology, and logistics, and constructing economic entities comprised of low-carbon enterprises, homes, buildings, and transportation. In terms of the current MDA energy consumption, mainly secondary energy represented by electricity is used, whereas the energy consumption contributed by high polluting fuels, such as raw coal, is less than 1%. Activity level data, covering industrial enterprises above the scale of the park from 2016–2021, were collected annually through field research and the distribution of questionnaires in the park.

### 2.2. Accounting Boundary and Scope

Clarifying and unifying accounting boundaries is required to assure the accuracy and completeness of CO_2_ emission accounting results [30]. All IPs have clear geographical boundaries; therefore, most existing studies are based on these geographical boundaries. However, with the development of the park, its actual area has expanded; therefore, the geographical boundary of the park has become difficult to define [7]. Moreover, although most enterprises are settled and operated within these boundaries, several enterprises registered in the park operate outside the park’s boundaries because of its preferential policies. Therefore, in an effort to accurately and comprehensively account for CO_2_ emissions in these IPs, the CO_2_ emissions inventory of this study included the CO_2_ emissions generated by all the enterprises registered in the park.

**Figure 1:**
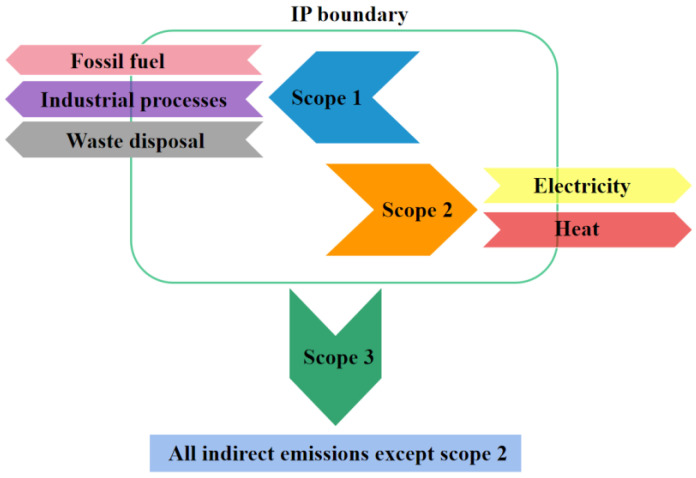
Accounting boundary and scopes of industrial park (IP) CO_2_ emissions.

To date, no IP emission scope standard has been established. Therefore, similar to previous studies, we concentrated on scope 1, 2 and 3 CO_2_ emissions of IPs based on the WBCSD- and WRI-defined GHG accounting scopes and the characteristics of the IPs. The accounting boundary and scope of IP CO_2_ emissions are shown in Figure 1. Scope 1 and 2 encompass the main CO_2_ emission sources in most IPs, and their calculation results are relatively clear and accurate, which is an important consideration in CO_2_ accounting. Information on scope 3 emissions does not have strong policy implications for reducing emissions in IPs and is very demanding in terms of data and accounting methods [25]. Consequentially, when calculating the CO_2_ emissions of IPs, measures should be adjusted in accordance with the local conditions and actual situation of the IPs, and important and related activities should be selected for accounting and reporting purposes.

### 2.3. Accounting Methods

#### 2.3.1. Energy Consumption

CO_2_ emissions from energy consumption can be classified as either direct or indirect. Specifically, direct emissions include fossil fuel combustion, and indirect emissions include electricity and heat consumption. In this study, biomass fuels were not considered because the amount of CO_2_ absorbed during plant growth is nearly equal to the amount of CO_2_ released by combustion; therefore, we believe that biomass fuels can generally achieve zero CO_2_ emission [31].

- Fossil fuel combustion

The energy-related CO_2_ emissions were calculated using Equation (1) as follows:

(1) $$CO_{2_{energy\;consumption}}=\overset{n}{\underset{i=1}{\sum }}\left(AD_{i}\times NCV_{i}\times CC_{i}\times COF_{i}\times \frac{44}{12}\right)$$

In this case, ${CO}_{2_{\mathrm{energy}\;\mathrm{consumption}}}$ is the CO_2_ emissions from energy consumption; *AD_i_* is the consumption of energy type *i*; *NCV_i_* is the net caloric value of fossil fuel *i*; *CC_i_* is the carbon content that represents the amount of CO_2_ emitted per unit of heat released; *COF_i_* is the oxygenation efficiency, which is defined as the fuel combustion ratio in boilers; and 44/12 is the CO_2_ gasification coefficient. Some research institutions, such as IPCC [9] and EEA [32], have supplied CO_2_ emissions factors (NCV, CC, and O) for fossil fuels. Accordingly, this study employed the CO_2_ emission factors that correspond the most with China’s environment.

- Electricity consumption

The electricity CO_2_ emissions considered in this study represented the CO_2_ emissions generated by the production of purchased electricity, which was included as scope 2 emissions. Moreover, the CO_2_ emissions generated by self-produced electricity in the park were included as scope 1 emissions. The electricity CO_2_ emissions of the park were calculated using Equation (2) as follows:

(2) $$CO_{2_{electricity}}=AD_{electricity}\times EF_{electricity}$$

In this case, *AD_electricity_* is the consumption of purchased electricity and *EF_electricity_* is the emission factor for electricity. *EF_electricity_* can refer to the emission factors of China’s regional power grid baseline, which is regularly published by the National Development and Reform Commission (NDRC).

- Heat consumption

In this study, the CO_2_ emissions from heat represented indirect emissions resulting from IPs purchasing heat from third parties outside the boundary. CO_2_ emissions from the heat produced in IPs were calculated as scope 1 emissions; therefore, the emission factors for purchased heat were not used. The relevant departments in China have not issued unified CO_2_ emissions factors for heat. Therefore, the total heat production, type of fuel consumed, and total fuel consumption provided in the energy balance sheets of provinces and autonomous regions of the *Energy Yearbook* were used to calculate the CO_2_ emission factors of purchased heat in each region.

(3) $$EF_{heat}=\frac{CE_{heat}}{Prod_{heat}}$$

In Equation (3), *EF_heat_* is the emission factor of heat, *CE_heat_* is the total CO_2_ emissions from fossil fuel inputs used for heating in the IP region, and *Prod_heat_* is the heat supply in the IP area. Heat supply CO_2_ emission factors should take priority over the calculated emission factors. Moreover, if insufficient data exists to calculate the emission factors, emission factors can be denoted as 0.11 tons of CO_2_/GJ.

#### 2.3.2. Industrial Process

The industrial process CO_2_ emissions inventory refers to CO_2_ emissions from chemical reaction or physical change processes other than the energy activities in industrial processes. For example, the production processes of cement, lime, calcium carbide, and steel all produce CO_2_. The CO_2_ emission inventory of industrial processes varies due to the large differences in leading industries and production processes of different IPs. Therefore, when formulating the emissions inventory of industrial processes, we used the carbon emission accounting standards of the corresponding industries for data statistics and CO_2_ emission calculations according to the park’s industrial structure and key industries. The detailed calculation methods refer to the GHG emission accounting methods and reporting guidelines of 24 industrial enterprises issued by NDRC [33,34,35]. Food processing and manufacturing and equipment manufacturing were used as the CO_2_ emission sources of the MDA industrial processes as follows:

(4) $$CO_{2_{industrial\;process}=}CO_{2_{food\;processing\;and\;manufacturing}}+CO_{2_{equipment\;manufacturing}}$$

(5) $$CO_{2_{food\;processing\;and\;manufacturing}}=AD_{carbonate}\times EF_{carbonate}$$

In the above equations, ${CO}_{2_{industrial\;process}}$ represents the CO_2_ emissions of the MDA industrial process, ${CO}_{2_{food\;processing\;and\;manufacturing}}$ represents the CO_2_ emissions of food processing and manufacturing in the MDA that was calculated, and ${CO}_{2_{equipment\;manufacturing}}$ represents the CO_2_ emissions of equipment manufacturing in the MDA as collected from several enterprises.

#### 2.3.3. Waste Disposal

Waste incineration also emits CO_2_. The waste types included municipal solid waste, hazardous waste, medical waste, and sewage sludge. CO_2_ emissions from waste incineration was calculated as follows:

(6) $$CO_{2_{waste\;incineration}}=\sum_{i}\left(IW_{i}\times CCW_{i}\times FCF_{i}\times EF_{i}\times \frac{44}{12}\right)$$

In this case, ${CO}_{2_{waste\;incineration}}$ refers to CO_2_ emissions from waste incineration, *IW_i_* refers to the incineration amount of type *i* waste, *CCW_i_* refers to the proportion of carbon content in type *i* waste, *FCF_i_* refers to the ratio of mineral carbon to total carbon in type *i* waste, and *EF_i_* refers to the combustion efficiency of the type *i* waste incinerator. We recommend the use of locally measured emission factors or those recommended by NDRC [36]. It should be noted that the CO_2_ emitted by IP waste treatment belongs to the scope 1 emissions; if the waste is treated outside the park, the generated CO_2_ emissions belong to the scope 3 emissions.

### 2.4. Uncertainty Analysis

**Figure 2:**
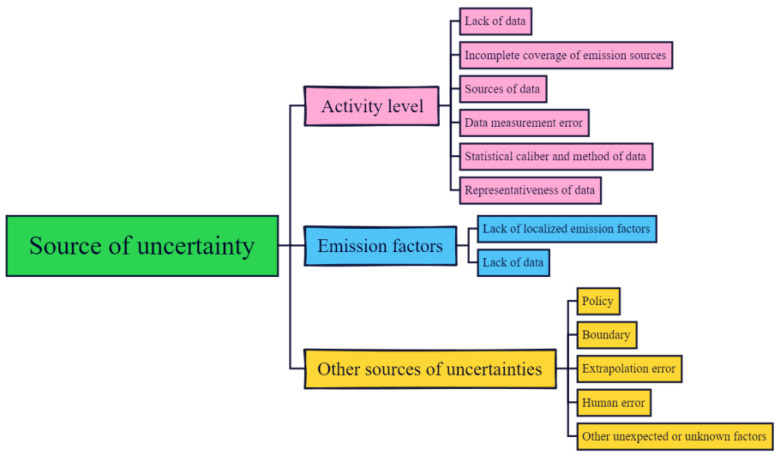
Uncertainty analysis framework of the CO_2_ emissions inventory in industrial parks (IPs).

Uncertainty analyses are important considerations when formulating CO_2_ emissions inventories [37,38,39]. As shown in Figure 2, we established the framework of uncertainty analyses for the qualitative evaluation of the uncertainty of IP CO_2_ emission inventories. The uncertainty of the inventory mainly originated as data and emission factor uncertainties. On this basis, we also considered the characteristics of the IPs themselves to allow a more accurate analysis of the inventory uncertainties. 

## 3. Results and Discussion

### 3.1. Total Emissions and Emissions Intensity

**Figure 3:**
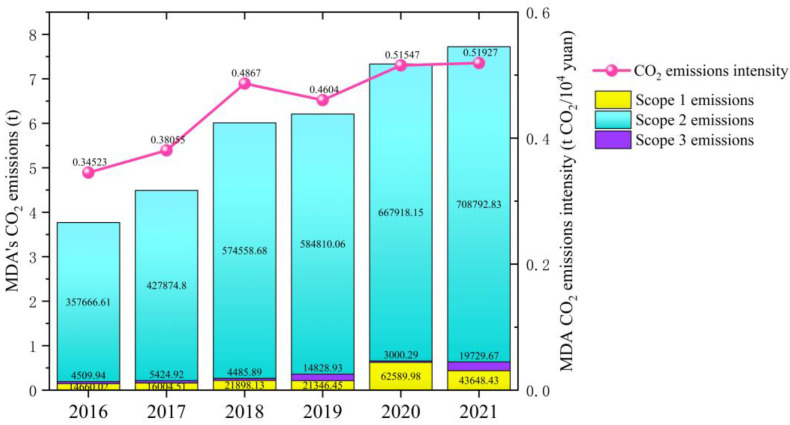
Total emissions and emissions intensity from 2016 to 2021.

The MDA CO_2_ emissions showed an accelerated upward trend in 2016–2021 (Figure 3), indicating that the MDA is still in the rapid development stage. From 2016 to 2021, the total amount of the MDA CO_2_ emissions increased by 395,334.36 tons, with an average annual increase of 15.43%. In 2021, the MDA CO_2_ emissions were 772,170.93 tons, which increased by 105% compared to that in 2016. Of these 2021 CO_2_ emissions, scope 1, 2, and 3 emissions accounted for 5.65%, 91.79%, and 2.56%, respectively. Throughout the study period, the scope 2 emissions were the largest, continually growing CO_2_ emission sources of the MDA, and their proportion remained at approximately 95%. Although the scope 1 emissions remained low, they increased over the past two years. Scope 3 emissions were the lowest of the MDA CO_2_ emissions and fluctuated upwards. Overall, the MDA energy structure is dominated by electricity and heat purchased outside the park, and clean energy is predominantly utilized throughout the park; however, non-renewable energy sources, such as raw coal, are still used. Although the consumption of non-renewable energy is small, its pollutants are the main atmospheric pollution sources in the park. In line with the changing CO_2_ emissions trend from 2016 to 2021, there was an overall upward trend in the CO_2_ emissions intensity. During this time, the CO_2_ emissions intensity increased by 0.174 t CO_2_/10^4^ yuan, with an average annual growth of 8.51%. In contrast to the rapid growth of CO_2_ emissions, the rise in CO_2_ intensity was relatively slow. Compared with other domestic IPs, the MDA CO_2_ emissions intensity remained low in recent years, indicating the efforts of the MDA as a low-carbon ecological demonstration park in the development of the low-carbon economy.

### 3.2. Industrial Energy Consumption Emissions by Sectors

**Figure 4:**
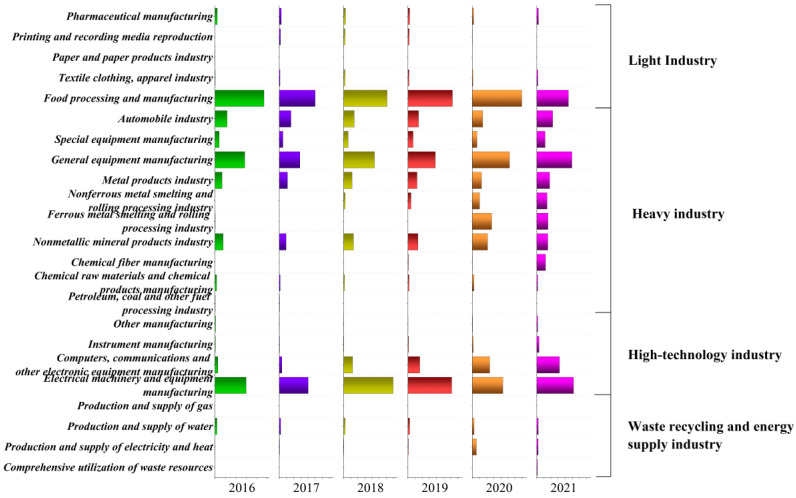
CO_2_ emissions by the Maanshan Economic and Technological Development Area (MDA) sectors from 2016 to 2021.

**Figure 5:**
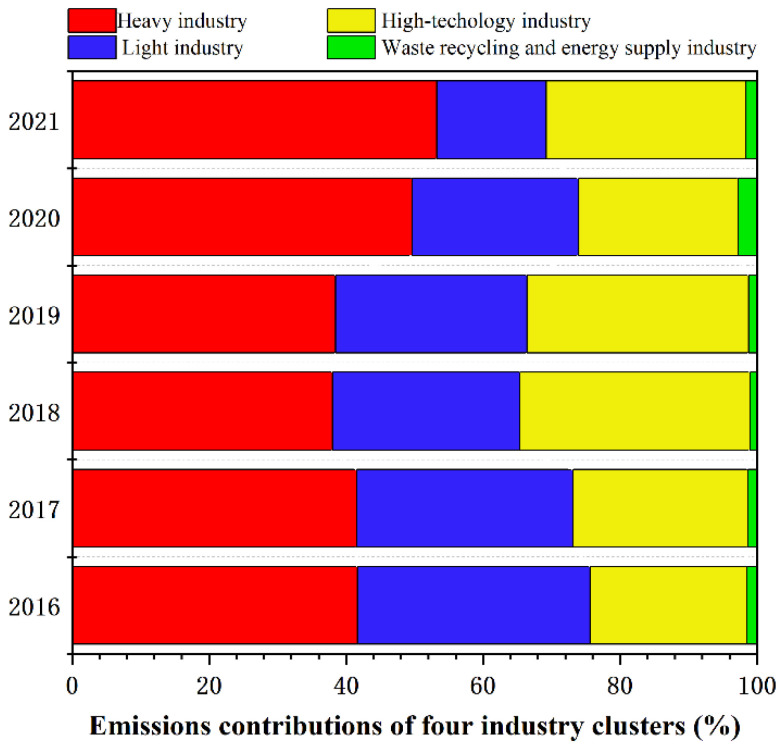
CO_2_ emissions contribution rates of four industry clusters from 2016 to 2021.

Industrial energy consumption emissions contributed more than 77% of the MDA CO_2_ emissions. Therefore, reducing industrial energy consumption emissions is the primary obstacle for the park to achieve carbon peak. To illustrate the relationship between the detailed industrial structure and the CO_2_ emissions of the MDA, we analyzed the MDA sectoral emissions (Figure 4). The MDA consists of 23 sector types, which we further classified into 4 clusters: light, heavy, high-technology, and waste recycling and energy supply industries. To better describe each cluster in the MDA, the CO_2_ emissions contribution rates from the 4 clusters were analyzed (Figure 5).

The changes in the MDA industrial structure were indicated by the changes in CO_2_ emissions across the sectors. It can be noted from Figure 4 and Figure 5 that the heavy industry CO_2_ emissions contributed the most to the MDA industrial energy consumption emissions, and its proportion increased from 41% in 2016 to 53% in 2021. General equipment manufacturing and the automobile industry were the main emission sources of the heavy industry, accounting for over 45% of the heavy industry CO_2_ emissions. This was followed by the light industry, which, in contrast with the heavy industry, showed an annual downward trend from 34% in 2016 to 16% in 2021. Food processing and manufacturing were the largest emissions source in the light industry, accounting for more than 90% of its CO_2_ emissions. Equipment manufacturing, automobile industry, and food processing and manufacturing are the three pillar industries of the MDA. Accordingly, these industries were the main MDA CO_2_ emission sources. Compared with other industries, the high-technology industry maintains a relatively stable contribution to CO_2_ emissions, with an annual average contribution rate of 28%. In recent years, the CO_2_ emissions of computers, communications, and other electronic equipment manufacturing grew rapidly, accounting for nearly 40% of the high-technology industry CO_2_ emissions. This indicated the rapid development and industrial advancements in the Yangtze River Delta and Pearl River Delta areas. Furthermore, the waste recycling and energy supply industry had the lowest CO_2_ emissions, indicating that its development is still in its infancy.

### 3.3. CO_2_ Flow Charts of 2016 and 2021

**Figure 6:**
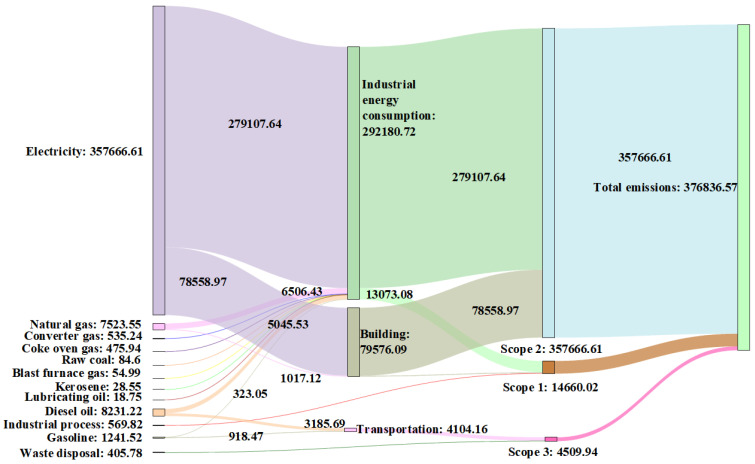
The Maanshan Economic and Technological Development Area (MDA) CO_2_ flow chart of 2016. Measurements are indicated in tons.

**Figure 7:**
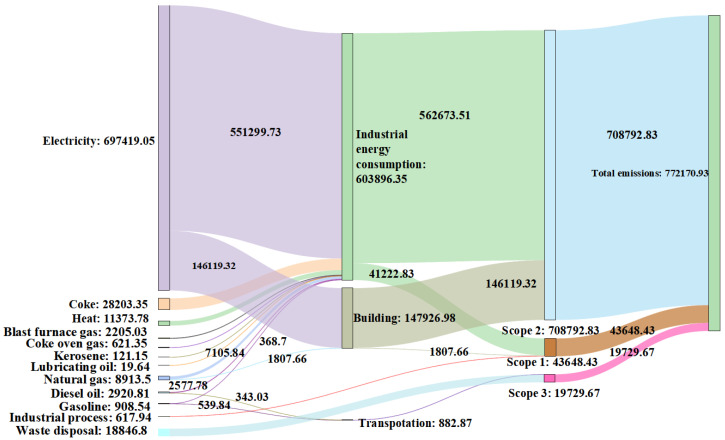
The Maanshan Economic and Technological Development Area (MDA) CO_2_ flow chart of 2021. Measurements are indicated in tons.

The MDA CO_2_ flow charts of 2016 (Figure 6) and 2021 (Figure 7) were constructed based on the calculated data and scope of CO_2_ emissions. Different emission sources are represented in different colors, and the width of the branches corresponds to the size of CO_2_ flow (Figure 6 and Figure 7).

The MDA CO_2_ flowthrough energy consumption, industrial processes, and waste disposal are shown on the left side of the charts. Among them, the CO_2_ emitted by energy consumption accounted for 99.9% of the total emissions. Evidently, electricity was the main source of the CO_2_ flow in the MDA, accounting for 95% and 90% of the total flow in 2016 and 2021, respectively. At least 77% of the total CO_2_ flowed into industrial energy consumption, in which the CO_2_ flow of electricity was the major source. The MDA energy structure was dominated by electricity rather than primary energy; however, the Anhui Province predominantly utilizes coal-fired electricity generation. Accordingly, the proportion of coal-fired power generation was as high as 90%. Therefore, the MDA still needs to strengthen the development and utilization of clean and renewable energy. The energy consumption CO_2_ subsequently flowed into three main parts, namely industrial energy consumption, buildings, and transportation. The percentages of every part in the total CO_2_ flows varied slightly in 2021 compared to those in 2016. Significantly, the 2016 CO_2_ flow percentage of electricity in industrial energy consumption decreased by 4% compared to that in 2021. Additionally, the heat and coke CO_2_ flowed into industrial energy consumption in 2021. The bottom-right areas in Figure 6 and Figure 7 indicate the CO_2_ flowing to the different scopes. In this case, it is apparent that the CO_2_ flow of scope 2 was much higher than that of scope 1 and 3. The scope 1 CO_2_ flow proportion of industrial energy consumption increased by 5.27% in 2021 compared to that in 2016, which was closely related to the extensive usage of coke during the 2021 industrial development of the MDA. Therefore, CO_2_ emissions can be greatly reduced by adjusting the CO_2_ flow of coke. Although the contribution of scope 3 emissions increased by only 1.3% in 2021 compared to that in 2016, in terms of emissions, it increased by a factor of 19. From the perspective of the emission scopes, the scope 2 emissions were the most significant in the park, and the proportion of its total emissions has remained stable at more than 91%. Nevertheless, the small contribution of scope 1 and 3 to the total emissions cannot be ignored.

### 3.4. Uncertainty Analyses

#### 3.4.1. Uncertainty in Activity Level Data

Emission factors are currently the most widely used carbon emission accounting method. In principle, activity level data and emission factors are selected for each emission source according to the CO_2_ emission inventory. The product of the activity data and emission factors serve as the estimated CO_2_ emission of the emission sources of interest. Therefore, the activity level and emission factors affect the uncertainty of the inventory. 

Insufficient data availability can result in errors between the calculated results and the real values. Therefore, due to the limited scope and data availability, carbon sinks of forests and wetlands were not estimated in this study. Moreover, due to the lack of waste data, the CO_2_ emissions from waste disposals were also not calculated. The activity level data used in this study were derived from statistical and park survey data, which were relatively reliable; however, some data may have either been incomplete or contained statistical errors. In addition, the IPCC classifications of fossil energy are not consistent with the Chinese energy statistics, and various statistical methods may be used to process IP data, resulting in an insufficient representation thereof. We calculated the traffic CO_2_ emissions in the park based on annual gas station sales. Although this approach facilitated data collection, it also reduced the representativeness of the data, thereby incorporating uncertainty in the traffic CO_2_ emission estimations. In summary, activity level data may influence the uncertainty of the CO_2_ emission inventory. 

#### 3.4.2. Uncertainty in Emission Factors

Local emission factors were used in the greatest possible extent during inventory preparation to reduce the uncertainty of the inventory. However, in the absence of local emission factors, the default values provided by IPCC were used for some fuels. Nevertheless, according to Liu et al. [40], Chinese coal emission factors are on average 40% lower than the default recommended by the IPCC; therefore, the results were somewhat uncertain. Furthermore, due to the limited availability of electricity emission factors for 2020 and 2021, we utilized the electricity emission factors of 2019 to calculate the 2020 and 2021 CO_2_ emissions from electricity. In addition, electricity is the main energy source used in the MDA; therefore, the electricity emission factors incorporated significant uncertainty in the inventory.

#### 3.4.3. Uncertainty in Other Sources

Aside from the activity levels and emission factors, numerous factors may incorporate uncertainty in the inventory.

In the IPs of China, several preferential policies typically exist; therefore, several enterprises conducting business activities outside the park choose to register inside the park. This results in the separation of enterprise registration and the actual location of its operation, which impedes the establishment of the IP boundary in CO_2_ emissions accounting. The majority of studies focus on the geographical boundary of IPs; however, considering the comprehensiveness of data, we suggest that the statistical data boundary of IPs should preferentially be used. Nevertheless, different departments may adopt different accounting boundaries and statistical calibers when utilizing statistical data boundaries, thereby incorporating uncertainty in the inventory when combined with existing errors and other unknown factors of uncertainty. In summary, some uncertainty exists in the preparation of the CO_2_ emissions inventory for the MDA.

### 3.5. Comparison of the Study Results with That of Previous Studies

Table 1 shows the comparison of the emission estimation results generated in this study with those of other parks. This demonstrated that the emissions of other parks were considerably higher than those of the MDA, which was mainly due to the relatively small size of the MDA. Moreover, the proportion of the MDA scope 2 emissions was much higher than those of other parks, denoting the excessive reliance of the MDA on heat and electricity purchased outside of the park. Scope 1 and 2, which are considered in all other studies, were the main emission sources in the IPs. However, significant differences exist in the content covered by scope 3 in various parks. The accuracy of scope 3 emission assessments depends on the data source, particularly when the actual emissions data are unavailable. Consequentially, limited previous studies considered the scope 3 emissions.

## 4. Conclusions and Policy Implication

### 4.1. Conclusions

Low-carbon development in IPs is an important consideration in the establishment of the carbon peak and neutrality in China. With the progressive development of IPs in China, more specific low-carbon policies should be designed at the park level to reduce the park emissions and achieve the peak carbon neutrality goal. By establishing the park level emissions inventory, the emissions characteristics can be elucidated, which is the first step toward reducing IP emissions.

In this study, we established a comprehensive CO_2_ emissions inventory for IPs that included scope 1, 2, and 3 emissions. Scope 1 emissions included all direct emissions that occurred within the park boundary. Scope 2 emissions included imported electricity and heat, whereas scope 3 emissions included indirect emissions other than those of scope 2. The calculation of scope 1 and 2 is particularly important, considering that they encompass the main CO_2_ emissions sources in most IPs. However, scope 3 calculations are challenging, owing to the limited IP data availability on scope 3 emissions, particularly in terms of the supply chains [41]. The accuracy of scope 3 emission assessments depends on the data source, which is difficult to recognize and obtain. By utilizing the MDA as an example, we calculated and identified the main sources of CO_2_ emissions by analyzing the park CO_2_ emissions inventory results from 2016 to 2021. This demonstrated that the MDA CO_2_ emissions grew annually and were calculated to be 772,170.93 tons in 2021. Moreover, we compared the emissions of the three scopes and found that scope 2 emissions were the main source of the total CO_2_ emissions, owing to the imported electricity utilized as the dominant energy source for industrial enterprises in the MDA. Industrial CO_2_ emissions were major contributors, with heavy industry contributing the most. However, this inventory still contained several uncertainties. Therefore, we established a framework to qualitatively analyze the uncertainty of the inventory from various aspects. To promote the facilitative role of the inventory in emission reduction activities, several measures can be considered to reduce uncertainties, such as adopting local emission factors and improving the availability of activity levels.

### 4.2. Policy Implication 

The increased CO_2_ emissions of the MDA are mainly caused by the advancement of economic aggregation and industrial structures. The main approach to reduce prospective emissions involves the adjustment of the industrial structure and reduction of the CO_2_ emission intensity of various industries. The reduction of CO_2_ emission intensity necessitates the improvement of energy efficiency and structure.

It is worth noting that the accounting method for GHG emissions in IPs has not been established. Therefore, it is crucial to standardize GHG accounting methods for IPs. As opposed to the GHG accounting methods of countries, cities, enterprises, and industries, IPs require the formulation of specific GHG accounting methods. In other words, the compilation of emission inventories for IPs needs to be tailored in accordance with the characteristics of the park. This paper fully considered the availability of statistical data in the park, and ensured the comprehensiveness and feasibility of the accounting method to the furthest possible extent while pursuing the integrity and accuracy of the accounting results. 

### 4.3. Future Works

Although data for scope 3 are difficult to obtain, scope 3 emissions cannot be ignored. Therefore, future studies should comprehensively include scope 3 emissions when constructing emissions inventories. We believe that our study may facilitate subsequent research and promote the preparation of the IP CO_2_ emissions inventory.

## Figures and Tables

**Table 1:** Greenhouse gas emissions from the Scope 1 to 3 of other parks.

Parks	Year	GHG Emissions/Mt			Proportion			Reference
		Scope 1	Scope 2	Scope 3	Scope 1	Scope 2	Scope 3	
Suzhou Industrial Park	2015	4.14	1.37	-	75.11%	24.89%	-	Yu et al. [25]
Nanchang High-tech Industrial Development Zone		0.16	0.90	-	15.12%	84.88%	-	
Laocheng Economic Development Zone		5.92	−4.64	-	462.34%	−362.34%	-	
Zhengzhou High-tech industrial Development Zone		0.05	0.58	-	8.35%	91.65%	-	
Yongcheng Economic and Technology Development Zone	2017	11.80	3.00	-	79.73%	20.27%	-	Zhang et al. [40]
203 National Economic Development Zones	2015	1016	71	108	85.02%	5.94%	9.04%	Yan et al. [7]
	2016	1087	8	114	89.91%	0.66%	9.43%	
	2017	1194	−8	121	91.35%	−0.61%	9.26%	
Maanshan Economic and Technological Development Area	2016	0.015	0.358	0.005	3.89%	94.91%	1.20%	This study
	2017	0.016	0.428	0.005	3.56%	95.23%	1.21%	
	2018	0.022	0.575	0.004	3.64%	95.61%	0.75%	
	2019	0.021	0.585	0.015	3.44%	94.17%	2.39%	
	2020	0.063	0.668	0.003	8.53%	91.06%	0.41%	
	2021	0.044	0.709	0.020	5.65%	91.79%	2.56%	

## Data Availability

Data are available from the authors upon request.
